# 7-Meth­oxy-1-(4-nitro­benzo­yl)naph­thalen-2-yl 4-nitro­benzoate

**DOI:** 10.1107/S1600536811027619

**Published:** 2011-07-16

**Authors:** Toyokazu Muto, Daichi Hijikata, Akiko Okamoto, Hideaki Oike, Noriyuki Yonezawa

**Affiliations:** a Department of Organic and Polymer Materials Chemistry, Tokyo University of Agriculture & Technology, Koganei, Tokyo 184-8588, Japan

## Abstract

In the title compound, C_25_H_16_N_2_O_8_, the dihedral angle between the naphthalene ring system and the benzene ring of the nitro­phenyl ketone unit is 82.64 (7)°. The bridging ester O—C(=O)—C plane makes dihedral angles of 42.12 (8) and 11.47 (9)°, respectively, with the naphthalene ring system and the benzene ring of the nitro­phenyl ester unit. In the crystal, two types of weak inter­molecular C—H⋯O inter­actions are observed.

## Related literature

For electrophilic aromatic substitution of naphthalene deriv­atives, see: Okamoto & Yonezawa (2009 ▶). For the structures of closely related compounds, see: Muto *et al.* (2010 ▶); Mitsui, Nakaema, Noguchi, Okamoto & Yonezawa (2008 ▶); Mitsui, Nakaema, Noguchi & Yonezawa (2008 ▶); Mitsui *et al.* (2009 ▶); Nagasawa *et al.* (2010 ▶); Watanabe *et al.* (2010 ▶).
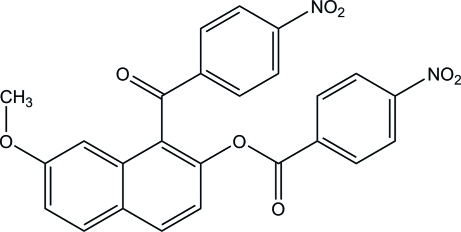


## Experimental

### 

#### Crystal data

C_25_H_16_N_2_O_8_
*M* *_r_* = 472.40
Triclinic, 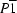

*a* = 7.30691 (15) Å
*b* = 10.2555 (2) Å
*c* = 14.7645 (3) Å
α = 84.750 (1)°
β = 86.278 (1)°
γ = 74.079 (1)°
*V* = 1058.57 (4) Å^3^
*Z* = 2
Cu *K*α radiation
μ = 0.95 mm^−1^
*T* = 193 K
0.60 × 0.20 × 0.10 mm

#### Data collection

Rigaku R-AXIS RAPID diffractometer
Absorption correction: numerical (*NUMABS*; Higashi, 1999 ▶) *T*_min_ = 0.599, *T*_max_ = 0.911
19272 measured reflections
3818 independent reflections
2769 reflections with *I* > 2σ(*I*)
*R*_int_ = 0.046

#### Refinement

*R*[*F* ^2^ > 2σ(*F* ^2^)] = 0.041
*wR*(*F* ^2^) = 0.124
*S* = 1.09
3818 reflections
319 parameters
H-atom parameters constrained
Δρ_max_ = 0.25 e Å^−3^
Δρ_min_ = −0.22 e Å^−3^

### 

Data collection: *PROCESS-AUTO* (Rigaku, 1998 ▶); cell refinement: *PROCESS-AUTO*; data reduction: *CrystalStructure* (Rigaku/MSC, 2004 ▶); program(s) used to solve structure: *SIR2004* (Burla *et al.*, 2005 ▶); program(s) used to refine structure: *SHELXL97* (Sheldrick, 2008 ▶); molecular graphics: *ORTEPIII* (Burnett & Johnson, 1996 ▶); software used to prepare material for publication: *SHELXL97*.

## Supplementary Material

Crystal structure: contains datablock(s) I, global. DOI: 10.1107/S1600536811027619/is2749sup1.cif

Structure factors: contains datablock(s) I. DOI: 10.1107/S1600536811027619/is2749Isup2.hkl

Supplementary material file. DOI: 10.1107/S1600536811027619/is2749Isup3.cml

Additional supplementary materials:  crystallographic information; 3D view; checkCIF report

## Figures and Tables

**Table 1:** Hydrogen-bond geometry (Å, °)

*D*—H⋯*A*	*D*—H	H⋯*A*	*D*⋯*A*	*D*—H⋯*A*
C6—H6⋯O7^i^	0.95	2.58	3.211 (3)	124
C23—H23⋯O4^ii^	0.95	2.55	3.435 (2)	154

Symmetry codes: (i) 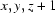; (ii) 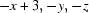.

**Fig. 1.**
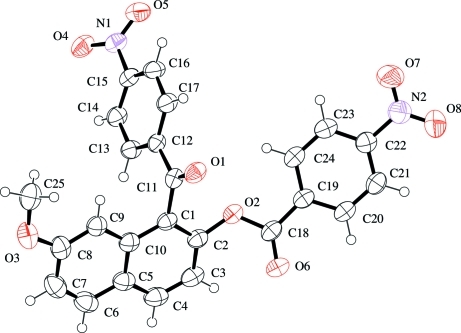
The molecular structure of the title compound with displacement ellipsoids at 50% probability.

**Fig. 2.**
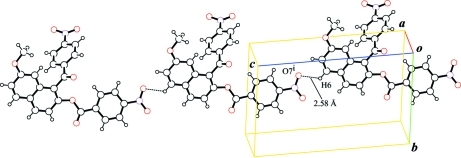
A partial packing diagram of the title compound, showing intermolecular C6—H6···O7i interactions [symmetry code: (i) x, y, 1 + z].

**Fig. 3.**
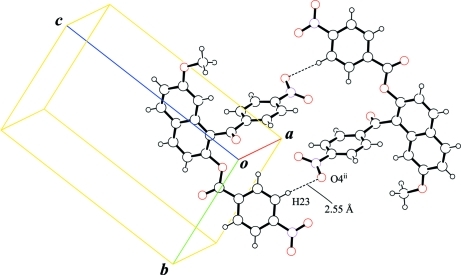
A partial packing diagram of the title compound, showing intermolecular C23—H23···O4ii interactions [symmetry code: (ii) 3 - x, -y, -z].

## supplementary crystallographic information

### Comment

In the course of our study on electrophilic aromatic aroylation of 2,7-dimethoxynaphthalene, *peri*-aroylnaphthalene compounds have proven to be formed regioselectively with the aid of suitable acidic mediators (Okamoto & Yonezawa, 2009). Recently, we have reported the crystal structures of several 1,8-diaroylated naphthalene homologues exemplified by 1,8-bis(4-methylbenzoyl)-2,7-dimethoxynaphthalene (Muto *et al.*, 2010). The aroyl groups at the 1,8-positions of the naphthalene rings in these compounds are twistedly connected in an almost perpendicular fashion, but the benzene ring moieties of the aroyl groups tilt slightly toward the *exo* sides of the naphthalene rings. The crystal structures of 1-monoaroylated naphthalene compounds such as 2,7-dimethoxy-1-(4-nitrobenzoyl)naphthalene (Watanabe *et al.*, 2010) also exhibit essentially the same non-coplanar structure as the 1,8-diaroylated naphthalenes. Furthermore, the crystal structures of several 1-monoaroylated naphthalene derivatives have revealed. For example, (2-hydroxy-7-methoxynaphthalen-1-yl)(4-methylphenyl)methanone (Nagasawa *et al.*, 2010) and (4-chlorophenyl)(2-hydroxy-7-methoxynaphthalen-1-yl)methanone (Mitsui, Nakaema, Noguchi & Yonezawa, 2008) prepared by regioselective demethylation form intramolecular hydrogen bond between the carbonyl group and the adjacent hydroxy one. Besides, (4-chlorobenzoyl)(2-ethoxy-7-methoxynaphthalen-1-yl)methanone (Mitsui *et al.*, 2009) has similar non-coplanar configuration to 1-(4-chlorobenzoyl)-2,7-dimethoxynaphthalene (Mitsui, Nakaema, Noguchi, Okamoto & Yonezawa, 2008). As a part of our continuous studies on the molecular structures of this kind of homologous molecules, the crystal structure of the title compound, 1-monoaroylated naphthalene bearing 4-nitrobenzoyloxy group at 2-position is discussed in this report.

The molecular structure of the title compound is displayed in Fig. 1. The benzene ring of nitrophenyl ketone moiety (C12–C17) is out of the plane of the naphthalene ring. The dihedral angle between the best planes of the benzene ring (C12–C17) and the naphthalene ring system (C1–C10) is 82.64 (7)° [C2–C1–C11–O1 torsion angle = -79.8 (2)°]. However, the carbonyl group (C11═O1) and the benzene ring (C12–C17) have almost coplanar configuration [O1–C11–C12–C17 torsion angle = 5.0 (2)°]. Besides, the dihedral angle between the benzene ring (C12–C17) and the nitro group plane (O4/N1/O5) is 29.35 (9)°. On the other hand, the benzene ring of nitrophenyl ester moiety (C19–C24) makes a rather small dihedral angle with naphthalene ring system (C1–C10) than that with the benzene ring of nitrophenyl ketone moiety (C12–C17), *i.e.*, 31.38 (7)°. Moreover, the dihedral angles of the benzene ring (C19–C24) and the naphthalene ring system (C1–C10) with the ester plane (O2—C18(═ O6)—C19) are 11.47 (9)° and 42.12 (8)°, respectively. The nitro group plane (O7/N2/O8) makes a small dihedral angle of 6.47 (10)° with the benzene ring (C19–C24). In the crystal structure, the molecular packing of the title compound is stabilized mainly by van der Waals interactions. The crystal packing is additionally stabilized by intermolecular C—H···O interaction between the oxygen atom (O7) of the nitro group in nitrophenyl ester and one hydrogen atom (H6) of the naphthalene ring of the adjacent molecule (C6—H6···O7^i^; Fig. 2 and Table 1). Moreover, there is also intermolecular C—H···O interaction between the oxygen atom (O4) of the nitro group in nitrophenyl ketone and one hydrogen atom (H23) of the nitrophenyl ester of the adjacent molecule (C23—H23···O4^ii^; Fig. 3 and Table 1).

### Experimental

To a 100 ml flask, 4-nitrobenzoyl chloride (17 mmol, 3.173 g), aluminium chloride (18.7 mmol, 2.495 g) and methylene chloride (21 ml) were placed and stirred at 273 K. To the reaction mixture thus obtained, 2,7-dimethoxynaphthalene (8.5 mmol, 1.599 g) was added. After the reaction mixture was stirred at 273 K for 60 h, it was poured into ice-cold water (10 ml). The aqueous layer was extracted with CHCl_3_ (10 ml × 3). The combined extracts were washed with 2 *M* aqueous NaOH followed by washing with brine. The organic layers thus obtained were dried over anhydrous MgSO_4_. The solvent was removed under reduced pressure to give cake. The crude product was purified by silica-gel chromatography from CHCl_3_. Yellow platelet single crystals suitable for X-ray diffraction were obtained by crystallization from ethanol (10 mg, 0.2% yield).

^1^H NMR δ (300 MHz, CDCl_3_); 3.75 (3*H*, s), 6.91 (1*H*, d, J = 2.4 Hz), 7.24 (1*H*, dd, J = 2.4, 9.0 Hz), 7.34 (1*H*, d, J = 8.4 Hz), 7.89 (1*H*, d, J = 9.0 Hz), 7.98 (2*H*, d, J = 9.0 Hz), 8.00 (2*H*, d, J = 8.4 Hz), 8.04 (1*H*, d, J = 9.0 Hz), 8.21 (2*H*, d, J = 8.4 Hz), 8.22 (2*H*, d, J = 9.3 Hz) p.p.m..

^13^C NMR δ (75 MHz, CDCl_3_); 55.32, 103.27, 118.37, 119.57, 123.65, 123.93, 125.45, 127.27, 130.14, 130.39, 131.00, 132.03, 132.54, 133.55, 141.98, 146.62, 150.55, 150.96, 159.37, 162.57, 194.18 p.p.m..

IR (KBr); 1746, 1679, 1619, 1525, 1349, 1234, 1208 cm^-1^

HRMS (m/*z*); [*M* + H]+ Calcd for C_25_H_17_N_2_O_8_, 473.0985; found, 473.0977.

m.p. = 452.0–454.0 K

### Refinement

All H atoms were found in a difference map and were subsequently refined as riding atoms, with C—H = 0.95 (aromatic) and 0.98 (methyl) Å, and with *U*_iso_(H) = 1.2*U*_eq_(C).

### Crystal data

**Table:**

C_25_H_16_N_2_O_8_	*Z* = 2
*M**_r_* = 472.40	*F*(000) = 488
Triclinic, *P*1	*D*_x_ = 1.482 Mg m^−^^3^
Hall symbol: -P 1	Melting point = 452.0–454.0 K
*a* = 7.30691 (15) Å	Cu *K*α radiation, λ = 1.54187 Å
*b* = 10.2555 (2) Å	Cell parameters from 13543 reflections
*c* = 14.7645 (3) Å	θ = 3.0–68.2°
α = 84.750 (1)°	µ = 0.95 mm^−^^1^
β = 86.278 (1)°	*T* = 193 K
γ = 74.079 (1)°	Platelet, yellow
*V* = 1058.57 (4) Å^3^	0.60 × 0.20 × 0.10 mm

### Data collection

**Table:**

Rigaku R-AXIS RAPID diffractometer	3818 independent reflections
Radiation source: rotating anode	2769 reflections with *I* > 2σ(*I*)
graphite	*R*_int_ = 0.046
Detector resolution: 10.00 pixels mm^-1^	θ_max_ = 68.2°, θ_min_ = 3.0°
ω scans	*h* = −8→8
Absorption correction: numerical (*NUMABS*; Higashi, 1999)	*k* = −12→12
*T*_min_ = 0.599, *T*_max_ = 0.911	*l* = −17→17
19272 measured reflections	

### Refinement

**Table:**

Refinement on *F*^2^	Secondary atom site location: difference Fourier map
Least-squares matrix: full	Hydrogen site location: inferred from neighbouring sites
*R*[*F*^2^ > 2σ(*F*^2^)] = 0.041	H-atom parameters constrained
*wR*(*F*^2^) = 0.124	*w* = 1/[σ^2^(*F*_o_^2^) + (0.066*P*)^2^] where *P* = (*F*_o_^2^ + 2*F*_c_^2^)/3
*S* = 1.09	(Δ/σ)_max_ < 0.001
3818 reflections	Δρ_max_ = 0.25 e Å^−^^3^
319 parameters	Δρ_min_ = −0.22 e Å^−^^3^
0 restraints	Extinction correction: *SHELXL97* (Sheldrick, 2008), Fc^*^=kFc[1+0.001xFc^2^λ^3^/sin(2θ)]^-1/4^
Primary atom site location: structure-invariant direct methods	Extinction coefficient: 0.0109 (9)

### Special details

**Table:**

Geometry. All e.s.d.'s (except the e.s.d. in the dihedral angle between two l.s. planes) are estimated using the full covariance matrix. The cell e.s.d.'s are taken into account individually in the estimation of e.s.d.'s in distances, angles and torsion angles; correlations between e.s.d.'s in cell parameters are only used when they are defined by crystal symmetry. An approximate (isotropic) treatment of cell e.s.d.'s is used for estimating e.s.d.'s involving l.s. planes.
Refinement. Refinement of *F*^2^ against ALL reflections. The weighted *R*-factor *wR* and goodness of fit *S* are based on *F*^2^, conventional *R*-factors *R* are based on *F*, with *F* set to zero for negative *F*^2^. The threshold expression of *F*^2^ > σ(*F*^2^) is used only for calculating *R*-factors(gt) *etc*. and is not relevant to the choice of reflections for refinement. *R*-factors based on *F*^2^ are statistically about twice as large as those based on *F*, and *R*- factors based on ALL data will be even larger.

### Fractional atomic coordinates and isotropic or equivalent isotropic displacement parameters (Å^2^)

**Table:**

	*x*	*y*	*z*	*U*_iso_*/*U*_eq_	
O1	0.72939 (15)	0.14056 (11)	0.14376 (8)	0.0481 (3)	
O2	0.81352 (16)	0.41705 (10)	0.13883 (8)	0.0441 (3)	
O3	0.7430 (2)	−0.05991 (14)	0.52467 (9)	0.0626 (4)	
O4	1.72999 (17)	−0.14620 (12)	0.21963 (9)	0.0572 (4)	
O5	1.69619 (17)	−0.13852 (12)	0.07413 (10)	0.0550 (4)	
O6	0.57830 (17)	0.61275 (11)	0.12164 (8)	0.0497 (3)	
O7	0.8971 (2)	0.37502 (16)	−0.32155 (10)	0.0792 (5)	
O8	0.7462 (2)	0.58713 (17)	−0.34326 (9)	0.0801 (5)	
N1	1.6332 (2)	−0.11728 (13)	0.15166 (11)	0.0460 (4)	
N2	0.8126 (2)	0.48522 (19)	−0.29361 (11)	0.0587 (4)	
C1	0.7879 (2)	0.26316 (16)	0.26282 (11)	0.0383 (4)	
C2	0.7743 (2)	0.39621 (16)	0.23280 (12)	0.0416 (4)	
C3	0.7412 (2)	0.50013 (18)	0.29209 (13)	0.0498 (5)	
H3	0.7355	0.5910	0.2698	0.060*	
C4	0.7176 (3)	0.46701 (19)	0.38256 (14)	0.0525 (5)	
H4	0.6969	0.5362	0.4237	0.063*	
C5	0.7230 (2)	0.33340 (18)	0.41736 (12)	0.0468 (4)	
C6	0.6962 (3)	0.2986 (2)	0.51139 (13)	0.0568 (5)	
H6	0.6712	0.3676	0.5528	0.068*	
C7	0.7053 (3)	0.1694 (2)	0.54338 (13)	0.0595 (5)	
H7	0.6882	0.1487	0.6069	0.071*	
C8	0.7402 (3)	0.0650 (2)	0.48347 (12)	0.0500 (5)	
C9	0.7681 (2)	0.09310 (17)	0.39186 (12)	0.0437 (4)	
H9	0.7933	0.0223	0.3518	0.052*	
C10	0.7594 (2)	0.22823 (17)	0.35694 (12)	0.0415 (4)	
C11	0.8441 (2)	0.15771 (15)	0.19458 (11)	0.0380 (4)	
C12	1.0499 (2)	0.07968 (15)	0.18749 (11)	0.0372 (4)	
C13	1.1798 (2)	0.09503 (16)	0.24817 (11)	0.0420 (4)	
H13	1.1372	0.1520	0.2968	0.050*	
C14	1.3706 (2)	0.02737 (16)	0.23747 (11)	0.0432 (4)	
H14	1.4597	0.0351	0.2793	0.052*	
C15	1.4283 (2)	−0.05105 (15)	0.16524 (12)	0.0403 (4)	
C16	1.3033 (2)	−0.06977 (17)	0.10467 (12)	0.0468 (4)	
H16	1.3475	−0.1255	0.0555	0.056*	
C17	1.1129 (2)	−0.00550 (16)	0.11758 (12)	0.0444 (4)	
H17	1.0236	−0.0196	0.0782	0.053*	
C18	0.6997 (2)	0.52340 (16)	0.08865 (12)	0.0412 (4)	
C19	0.7424 (2)	0.51149 (16)	−0.01015 (12)	0.0405 (4)	
C20	0.6551 (2)	0.62210 (17)	−0.06911 (13)	0.0449 (4)	
H20	0.5785	0.7031	−0.0451	0.054*	
C21	0.6792 (2)	0.61456 (17)	−0.16166 (13)	0.0468 (5)	
H21	0.6210	0.6898	−0.2022	0.056*	
C22	0.7898 (2)	0.49528 (18)	−0.19427 (12)	0.0451 (4)	
C23	0.8803 (2)	0.38428 (18)	−0.13809 (13)	0.0475 (5)	
H23	0.9570	0.3038	−0.1627	0.057*	
C24	0.8563 (2)	0.39339 (17)	−0.04519 (12)	0.0448 (4)	
H24	0.9177	0.3187	−0.0050	0.054*	
C25	0.7807 (3)	−0.1711 (2)	0.46867 (14)	0.0637 (6)	
H25A	0.7773	−0.2542	0.5064	0.076*	
H25B	0.9069	−0.1831	0.4383	0.076*	
H25C	0.6838	−0.1525	0.4228	0.076*	

### Atomic displacement parameters (Å^2^)

**Table:**

	*U*^11^	*U*^22^	*U*^33^	*U*^12^	*U*^13^	*U*^23^
O1	0.0423 (7)	0.0422 (7)	0.0595 (8)	−0.0068 (6)	−0.0133 (6)	−0.0083 (6)
O2	0.0433 (7)	0.0352 (6)	0.0505 (7)	−0.0052 (5)	−0.0038 (5)	−0.0019 (5)
O3	0.0694 (9)	0.0699 (9)	0.0509 (8)	−0.0263 (7)	−0.0071 (7)	0.0083 (7)
O4	0.0444 (8)	0.0506 (8)	0.0703 (9)	−0.0003 (6)	−0.0121 (7)	−0.0036 (6)
O5	0.0492 (8)	0.0476 (7)	0.0659 (9)	−0.0096 (6)	0.0103 (7)	−0.0117 (6)
O6	0.0436 (7)	0.0381 (7)	0.0632 (8)	−0.0028 (6)	−0.0013 (6)	−0.0088 (6)
O7	0.0857 (11)	0.0800 (11)	0.0672 (10)	−0.0038 (9)	−0.0163 (8)	−0.0293 (8)
O8	0.0850 (11)	0.0858 (11)	0.0592 (9)	−0.0083 (9)	−0.0114 (8)	0.0088 (8)
N1	0.0425 (9)	0.0341 (8)	0.0600 (10)	−0.0082 (6)	0.0006 (8)	−0.0052 (7)
N2	0.0486 (10)	0.0684 (11)	0.0592 (11)	−0.0123 (9)	−0.0103 (8)	−0.0101 (9)
C1	0.0298 (9)	0.0371 (9)	0.0473 (10)	−0.0061 (7)	−0.0052 (7)	−0.0066 (7)
C2	0.0346 (9)	0.0393 (9)	0.0489 (10)	−0.0053 (7)	−0.0041 (8)	−0.0060 (8)
C3	0.0452 (11)	0.0389 (9)	0.0642 (13)	−0.0061 (8)	−0.0076 (9)	−0.0110 (9)
C4	0.0468 (11)	0.0499 (11)	0.0606 (12)	−0.0070 (9)	−0.0056 (9)	−0.0201 (9)
C5	0.0356 (10)	0.0528 (11)	0.0512 (11)	−0.0068 (8)	−0.0050 (8)	−0.0136 (9)
C6	0.0481 (12)	0.0726 (14)	0.0499 (12)	−0.0116 (10)	−0.0045 (9)	−0.0192 (10)
C7	0.0546 (12)	0.0812 (15)	0.0444 (11)	−0.0202 (11)	−0.0055 (9)	−0.0062 (10)
C8	0.0419 (10)	0.0619 (12)	0.0476 (11)	−0.0170 (9)	−0.0075 (8)	0.0016 (9)
C9	0.0359 (9)	0.0471 (10)	0.0474 (10)	−0.0090 (8)	−0.0056 (8)	−0.0044 (8)
C10	0.0301 (9)	0.0464 (10)	0.0474 (10)	−0.0072 (7)	−0.0059 (7)	−0.0071 (8)
C11	0.0381 (9)	0.0345 (8)	0.0422 (9)	−0.0109 (7)	−0.0078 (8)	0.0011 (7)
C12	0.0389 (9)	0.0312 (8)	0.0406 (9)	−0.0081 (7)	−0.0048 (7)	−0.0011 (7)
C13	0.0379 (10)	0.0419 (9)	0.0453 (10)	−0.0071 (8)	−0.0027 (7)	−0.0087 (8)
C14	0.0394 (10)	0.0422 (9)	0.0481 (11)	−0.0091 (8)	−0.0085 (8)	−0.0056 (8)
C15	0.0357 (9)	0.0307 (8)	0.0516 (10)	−0.0049 (7)	−0.0012 (8)	−0.0014 (7)
C16	0.0460 (11)	0.0414 (10)	0.0502 (11)	−0.0044 (8)	−0.0004 (8)	−0.0133 (8)
C17	0.0422 (10)	0.0423 (9)	0.0481 (10)	−0.0068 (8)	−0.0097 (8)	−0.0089 (8)
C18	0.0361 (9)	0.0309 (8)	0.0582 (11)	−0.0109 (7)	−0.0063 (8)	−0.0021 (8)
C19	0.0351 (9)	0.0337 (9)	0.0539 (11)	−0.0102 (7)	−0.0041 (8)	−0.0053 (8)
C20	0.0400 (10)	0.0338 (9)	0.0597 (12)	−0.0070 (7)	−0.0057 (8)	−0.0042 (8)
C21	0.0402 (10)	0.0423 (10)	0.0576 (12)	−0.0104 (8)	−0.0098 (8)	0.0016 (8)
C22	0.0377 (10)	0.0490 (10)	0.0500 (11)	−0.0123 (8)	−0.0063 (8)	−0.0062 (8)
C23	0.0409 (10)	0.0419 (10)	0.0596 (12)	−0.0079 (8)	−0.0043 (8)	−0.0112 (8)
C24	0.0398 (10)	0.0364 (9)	0.0566 (11)	−0.0070 (7)	−0.0071 (8)	−0.0018 (8)
C25	0.0671 (14)	0.0584 (13)	0.0665 (14)	−0.0216 (11)	−0.0093 (11)	0.0094 (10)

### Geometric parameters (Å, °)

**Table:**

O1—C11	1.2181 (17)	C9—C10	1.420 (2)
O2—C18	1.3654 (19)	C9—H9	0.9500
O2—C2	1.407 (2)	C11—C12	1.499 (2)
O3—C8	1.362 (2)	C12—C17	1.387 (2)
O3—C25	1.425 (2)	C12—C13	1.395 (2)
O4—N1	1.2319 (17)	C13—C14	1.382 (2)
O5—N1	1.2214 (18)	C13—H13	0.9500
O6—C18	1.2016 (19)	C14—C15	1.369 (2)
O7—N2	1.224 (2)	C14—H14	0.9500
O8—N2	1.222 (2)	C15—C16	1.380 (2)
N1—C15	1.474 (2)	C16—C17	1.375 (2)
N2—C22	1.475 (2)	C16—H16	0.9500
C1—C2	1.374 (2)	C17—H17	0.9500
C1—C10	1.419 (2)	C18—C19	1.481 (2)
C1—C11	1.502 (2)	C19—C24	1.390 (2)
C2—C3	1.401 (2)	C19—C20	1.395 (2)
C3—C4	1.360 (3)	C20—C21	1.373 (2)
C3—H3	0.9500	C20—H20	0.9500
C4—C5	1.410 (2)	C21—C22	1.377 (2)
C4—H4	0.9500	C21—H21	0.9500
C5—C6	1.416 (2)	C22—C23	1.381 (2)
C5—C10	1.420 (2)	C23—C24	1.380 (2)
C6—C7	1.351 (3)	C23—H23	0.9500
C6—H6	0.9500	C24—H24	0.9500
C7—C8	1.410 (3)	C25—H25A	0.9800
C7—H7	0.9500	C25—H25B	0.9800
C8—C9	1.371 (2)	C25—H25C	0.9800
			
C18—O2—C2	120.25 (13)	C13—C12—C11	121.03 (15)
C8—O3—C25	117.74 (14)	C14—C13—C12	120.00 (16)
O5—N1—O4	124.40 (15)	C14—C13—H13	120.0
O5—N1—C15	118.15 (15)	C12—C13—H13	120.0
O4—N1—C15	117.45 (15)	C15—C14—C13	118.59 (15)
O8—N2—O7	123.76 (19)	C15—C14—H14	120.7
O8—N2—C22	118.23 (17)	C13—C14—H14	120.7
O7—N2—C22	118.01 (17)	C14—C15—C16	122.86 (16)
C2—C1—C10	119.72 (15)	C14—C15—N1	118.25 (15)
C2—C1—C11	118.15 (15)	C16—C15—N1	118.89 (16)
C10—C1—C11	122.03 (14)	C17—C16—C15	118.09 (16)
C1—C2—C3	122.62 (17)	C17—C16—H16	121.0
C1—C2—O2	114.39 (14)	C15—C16—H16	121.0
C3—C2—O2	122.75 (15)	C16—C17—C12	120.77 (15)
C4—C3—C2	117.97 (17)	C16—C17—H17	119.6
C4—C3—H3	121.0	C12—C17—H17	119.6
C2—C3—H3	121.0	O6—C18—O2	123.46 (17)
C3—C4—C5	122.17 (17)	O6—C18—C19	125.18 (15)
C3—C4—H4	118.9	O2—C18—C19	111.34 (15)
C5—C4—H4	118.9	C24—C19—C20	119.88 (17)
C4—C5—C6	122.42 (17)	C24—C19—C18	122.59 (15)
C4—C5—C10	119.49 (17)	C20—C19—C18	117.44 (15)
C6—C5—C10	118.08 (16)	C21—C20—C19	120.39 (16)
C7—C6—C5	121.38 (18)	C21—C20—H20	119.8
C7—C6—H6	119.3	C19—C20—H20	119.8
C5—C6—H6	119.3	C20—C21—C22	118.35 (16)
C6—C7—C8	120.66 (18)	C20—C21—H21	120.8
C6—C7—H7	119.7	C22—C21—H21	120.8
C8—C7—H7	119.7	C21—C22—C23	122.92 (17)
O3—C8—C9	125.24 (17)	C21—C22—N2	118.69 (16)
O3—C8—C7	114.50 (17)	C23—C22—N2	118.39 (16)
C9—C8—C7	120.26 (17)	C24—C23—C22	118.21 (17)
C8—C9—C10	119.91 (17)	C24—C23—H23	120.9
C8—C9—H9	120.0	C22—C23—H23	120.9
C10—C9—H9	120.0	C23—C24—C19	120.22 (16)
C1—C10—C9	122.34 (15)	C23—C24—H24	119.9
C1—C10—C5	117.96 (15)	C19—C24—H24	119.9
C9—C10—C5	119.70 (16)	O3—C25—H25A	109.5
O1—C11—C12	121.01 (15)	O3—C25—H25B	109.5
O1—C11—C1	121.49 (14)	H25A—C25—H25B	109.5
C12—C11—C1	117.41 (13)	O3—C25—H25C	109.5
C17—C12—C13	119.59 (15)	H25A—C25—H25C	109.5
C17—C12—C11	119.35 (14)	H25B—C25—H25C	109.5
			
C10—C1—C2—C3	2.9 (2)	O1—C11—C12—C13	−177.25 (14)
C11—C1—C2—C3	−173.59 (14)	C1—C11—C12—C13	6.3 (2)
C10—C1—C2—O2	177.35 (13)	C17—C12—C13—C14	1.1 (2)
C11—C1—C2—O2	0.9 (2)	C11—C12—C13—C14	−176.67 (14)
C18—O2—C2—C1	135.88 (14)	C12—C13—C14—C15	1.7 (2)
C18—O2—C2—C3	−49.7 (2)	C13—C14—C15—C16	−2.6 (2)
C1—C2—C3—C4	−1.5 (3)	C13—C14—C15—N1	176.96 (14)
O2—C2—C3—C4	−175.56 (15)	O5—N1—C15—C14	−150.67 (15)
C2—C3—C4—C5	−0.9 (3)	O4—N1—C15—C14	28.6 (2)
C3—C4—C5—C6	−179.22 (16)	O5—N1—C15—C16	28.9 (2)
C3—C4—C5—C10	1.8 (3)	O4—N1—C15—C16	−151.83 (15)
C4—C5—C6—C7	−178.77 (17)	C14—C15—C16—C17	0.6 (3)
C10—C5—C6—C7	0.2 (3)	N1—C15—C16—C17	−179.02 (14)
C5—C6—C7—C8	−0.7 (3)	C15—C16—C17—C12	2.4 (3)
C25—O3—C8—C9	0.9 (3)	C13—C12—C17—C16	−3.2 (2)
C25—O3—C8—C7	−178.95 (16)	C11—C12—C17—C16	174.59 (14)
C6—C7—C8—O3	−179.10 (17)	C2—O2—C18—O6	9.5 (2)
C6—C7—C8—C9	1.0 (3)	C2—O2—C18—C19	−168.93 (12)
O3—C8—C9—C10	179.26 (16)	O6—C18—C19—C24	−166.57 (15)
C7—C8—C9—C10	−0.9 (3)	O2—C18—C19—C24	11.9 (2)
C2—C1—C10—C9	178.82 (14)	O6—C18—C19—C20	10.0 (2)
C11—C1—C10—C9	−4.9 (2)	O2—C18—C19—C20	−171.59 (13)
C2—C1—C10—C5	−1.8 (2)	C24—C19—C20—C21	0.7 (2)
C11—C1—C10—C5	174.53 (13)	C18—C19—C20—C21	−175.95 (14)
C8—C9—C10—C1	179.79 (14)	C19—C20—C21—C22	0.5 (2)
C8—C9—C10—C5	0.4 (2)	C20—C21—C22—C23	−1.3 (3)
C4—C5—C10—C1	−0.5 (2)	C20—C21—C22—N2	178.58 (14)
C6—C5—C10—C1	−179.47 (14)	O8—N2—C22—C21	6.3 (2)
C4—C5—C10—C9	178.94 (15)	O7—N2—C22—C21	−173.67 (15)
C6—C5—C10—C9	−0.1 (2)	O8—N2—C22—C23	−173.82 (16)
C2—C1—C11—O1	−79.86 (19)	O7—N2—C22—C23	6.2 (2)
C10—C1—C11—O1	103.77 (18)	C21—C22—C23—C24	0.8 (3)
C2—C1—C11—C12	96.63 (17)	N2—C22—C23—C24	−179.08 (15)
C10—C1—C11—C12	−79.75 (18)	C22—C23—C24—C19	0.5 (2)
O1—C11—C12—C17	5.0 (2)	C20—C19—C24—C23	−1.2 (2)
C1—C11—C12—C17	−171.52 (14)	C18—C19—C24—C23	175.27 (14)

### Hydrogen-bond geometry (Å, °)

**Table:**

*D*—H···*A*	*D*—H	H···*A*	*D*···*A*	*D*—H···*A*
C6—H6···O7^i^	0.95	2.58	3.211 (3)	124
C23—H23···O4^ii^	0.95	2.55	3.435 (2)	154

Symmetry codes: (i) *x*, *y*, *z*+1; (ii) −*x*+3, −*y*, −*z*.
